# Interplay between microRNAs, Serum Proprotein Convertase Subtilisin/Kexin Type 9 (PCSK9), and Lipid Parameters in Patients with Very High Lipoprotein(a) Treated with PCSK9 Inhibitors

**DOI:** 10.3390/genes14030632

**Published:** 2023-03-03

**Authors:** Tina Levstek, Tina Karun, Andreja Rehberger Likozar, Miran Šebeštjen, Katarina Trebušak Podkrajšek

**Affiliations:** 1 Laboratory for Translational Medical Biochemistry, Institute of Biochemistry and Molecular Genetics, Faculty of Medicine, University of Ljubljana, Vrazov trg 2, 1000 Ljubljana, Slovenia; 2 Clinical Institute for Special Laboratory Diagnostics, University Children’s Hospital, University Medical Centre Ljubljana, Vrazov trg 1, 1000 Ljubljana, Slovenia; 3 Department of Vascular Diseases, University Medical Centre Ljubljana, Zaloška cesta 7, 1000 Ljubljana, Slovenia; 4 Department of Cardiology, University Medical Centre Ljubljana, Zaloška cesta 7, 1000 Ljubljana, Slovenia; 5 Department of Internal Medicine, Faculty of Medicine, University of Ljubljana, Zaloška cesta 7, 1000 Ljubljana, Slovenia

**Keywords:** cardiovascular disease, PCSK9, microRNA, lipoprotein(a), PCSK9 inhibitors, lipid parameters, biomarker

## Abstract

Proprotein convertase subtilisin/kexin type 9 (PCSK9) has an important function in the regulation of lipid metabolism. PCSK9 reduces hepatic low-density lipoprotein receptors, thereby increasing low-density lipoprotein cholesterol levels. However, its regulation remains to be elucidated, including post-transcriptional regulation by microRNAs (miRNAs). We aimed to explore the interplay between miRNAs, total serum PCSK9, and lipids during treatment with PCSK9 inhibitors. A total of 64 patients with stable coronary artery disease and very high lipoprotein(a) levels and 16 sex- and age-matched control subjects were enrolled. Patients received a PCSK9 inhibitor (evolocumab or alirocumab). Total serum PCSK9 levels were measured by immunoassay. RNA was isolated from plasma using magnetic beads, and expression of selected miRNAs was analyzed by quantitative PCR. Total serum PCSK9 levels were significantly higher in control subjects compared with patients. After 6 months of treatment with PCSK9 inhibitors, total serum PCSK9 levels increased significantly. The expression of miR-191-5p was significantly lower, and the expression of miR-224-5p and miR-483-5p was significantly higher in patients compared with control subjects. Using linear regression, the expression of miR-483-5p significantly predicted the serum PCSK9 level at baseline. After the 6-month period of therapy, the expression of miR-191-5p and miR-483-5p significantly increased. Our results support a role for miR-483-5p in regulating circulating PCSK9 *in vivo*. The difference in expression of miR-191-5p, miR-224-5p, and miR-337-3p between patients and control subjects suggests their possible role in the pathogenesis of coronary artery disease.

## 1. Introduction

The World Health Organization estimates that about one-third of all deaths worldwide are accounted to cardiovascular disease (CVD). The leading cause of CVD is atherosclerosis [1]. Elevated levels of low-density lipoprotein cholesterol (LDL-C) are casually associated with atherosclerotic CVDs and are the most important modifiable factor in the prevention of atherosclerosis and cardiovascular events [2]. Lipoprotein (a) (Lp(a)) is an independent risk factor for coronary artery disease (CAD), regardless of LDL-C levels before and also after its reduction, especially with statins [3]. The structure of Lp(a) resembles LDL-C in the composition of the lipid core and presence of apolipoprotein B (apoB). However, it also consists of the unique glycoprotein apolipoprotein(a) (apo(a)), which is bound to the apolipoprotein B100 moiety of an LDL-like particle via a disulfide bond [4,5]. Apo(a) is encoded by the *LPA* gene, and variants in this gene are responsible for as much as 90% of the variability in circulating Lp(a) levels [6].

Proprotein convertase subtilisin/kexin type 9 (PCSK9) is a soluble serine protease expressed predominantly in the liver and is considered a fundamental regulator of lipid metabolism [7]. When PCSK9 bound to LDL-C interacts with the LDL receptor (LDLR), the catalytic domain of PCSK9 interacts with LDLR. After endocytosis, the affinity of the interaction between PCSK9 and LDLR increases under acidic conditions, preventing LDLR from recycling. Instead, the complex is directed to the lysosome, where both are degraded. A lower abundance of LDLR on the surface of hepatocytes results in higher levels of LDL-C in plasma [8,9,10]. PCSK9 may also promote the degradation of LDLR through an intracellular pathways reported in cultured cells [11]. Beyond its role in cholesterol homeostasis, PCSK9 mediates inflammatory responses by binding to Toll-like receptors (TLRs) and promotes platelet activation and thrombosis by binding to scavenger receptor class B (SR-B) receptors. In addition, binding to low-density lipoprotein receptor-related protein 1 (LRP1), apolipoprotein E receptor-2 (ApoER2), very-low-density lipoprotein receptor (VLDLR), and other receptors promotes vascular endothelial hyperplasia and increases lipoprotein levels [12]. *PCSK9* loss-of-function variants have been associated with a reduction in circulating LDL-C levels and risk of coronary heart disease [13]. In the last decade, several monoclonal antibodies specifically targeting PCSK9 have been introduced in the clinical practice [14]. PCSK9 inhibitors bind circulating PCSK9, preventing its binding to LDLR and thus allowing its recycling. PCSK9 inhibitors reduce LDL-C levels by approximately 40–65% when used in conjunction with statins. Moreover, unlike statins, PCSK9 also reduces Lp(a) levels by 25–30% [15,16].

Nevertheless, the understanding of the pathways regulating PCSK9 availability is insufficient. Post-transcriptional regulation by microRNAs (miRNAs) is of particular interest because they are small, single-stranded, non-coding RNAs known to regulate gene expression by degrading messenger RNAs or inhibiting their translation [17]. Accumulating evidence suggests that miRNAs play a crucial role in cholesterol metabolism and in the development and progression of atherosclerosis through their involvement in endothelial integrity, monocyte/macrophage recruitment, and vascular smooth muscle and inflammatory cell function [18,19,20]. One of the most intensively studied miRNA implicated in the regulation of lipid homeostasis is miR-33. miR-33 is a family of sterol-sensitive miRNAs. They are involved in the regulation of very-low-density lipoprotein cholesterol (VLDL-C), LDL-C, and high-density lipoprotein cholesterol (HDL-C) metabolism, HDL-C uptake, cholesterol storage, transport, excretion, and efflux [21]. Understanding the regulation of PCSK9 by miRNAs could lead to the discovery of new biomarkers and provide new insights into the pathophysiology of atherosclerosis. The aim of the present study was to investigate miRNA expressions and circulating PCSK9 levels in patients with stable phase of CAD at least 6 months after acute myocardial infarction and very high Lp(a) levels and in control subjects. We also aimed to elucidate the relationship between serum PCSK9, miRNAs expression and lipid parameters after the patients were treated with PCSK9 inhibitors.

## 2. Materials and Methods

### 2.1. Study Participants

We included 64 patients and additional 16 control subjects matched for sex and age. Patients were in the stable phase of CAD at least 6 months after acute myocardial infarction. The inclusion criteria for patients were premature myocardial infarction before 50 years of age and an Lp(a) level of more than 1000 mg/L or an Lp(a) level of more than 600 mg/L and an LDL-C level of more than 2.6 mmol/L. Patients were receiving state-of-the-art therapy that had not been changed for at least 2 months before enrollment in the study. They received a statin at a maximally tolerated dose plus ezetimibe, if needed. They also received β-blockers, antiplatelet agents, and angiotensin-converting enzyme inhibitors. The following exclusion criteria applied: elevated liver transaminases enzyme activity (more than three times above reference values) and severe renal dysfunction (serum creatinine more than 200 mmol/L). In addition, patients were excluded if they had acute illness in the last 6 weeks. Control subjects had no history of CVD, no hypercholesterolemia, and an Lp(a) level less than 300 mg/L.

After enrollment in the study, blood samples were collected, and patients underwent clinical and laboratory examinations [22]. They were divided into two groups regarding treatment with PCSK9 inhibitors. The first group received placebo for 6 months. This was followed by subcutaneous treatment with a PCSK9 inhibitor, namely alirocumab at a dose of 150 mg or evolocumab at a dose of 140 mg, every two weeks. The second group, however, received the PCSK9 inhibitor immediately after enrollment in the study. Laboratory parameters were determined at baseline and later after the placebo phase, and after treatment with PCSK9 inhibitors. The Slovenian National Ethics Committee endorsed the protocol of the study (No. 0120-357/2018/8 and No. 0120-317/2021/3). All participants were informed about the study protocol and signed an informed consent prior to the enrollment, as recommended by the Declaration of Helsinki.

### 2.2. Biochemical Analysis

Blood for laboratory analysis was drawn in the morning after 12 hours of fasting. We collected samples from the antecubital vein into 5 mL vacuum-sealed tubes containing clot activator (Vacutube; LT Burnik, Skaručna, Slovenia). We centrifuged the blood at 2000× *g* for 15 min to separate the serum. In fresh serum samples, we measured total cholesterol, triglycerides, HDL-C, apolipoprotein A1 (apoA1), and apoB by standard colorimetric or immunologic assays. We used an automated biochemical analyzer (Fusion 5.1; Ortho-Clinical Diagnostics, Raritan, NJ, USA). The same biochemical analyzer was used to determine Lp(a) with the Denka reagent (Randox, Crumlin, UK). Because of the apo(a)-isoform-insensitive antibodies used in the reagent, the bias associated with apo(a) size is minimal. The Friedewald formula [23] was used to calculate LDL-C.

### 2.3. Measurement of Serum PCSK9 Levels

Total PCSK9 was measured in serum samples using a sandwich enzyme-linked immunosorbent assay (ELISA) (Human Proprotein Convertase 9/PCSK9 Quantikine^®^ ELISA Kit, R&D Systems, Minneapolis, MN, USA) on a Sunrise microplate reader and Magellan software (Tecan, Männedorf, Switzerland) as instructed by the manufacturer. Sensitivity and assay range provided by the manufacturer were 0.219 ng/mL and 0.6–40 ng/mL, respectively. Samples were diluted 1:20 with calibrator diluent. Samples exceeding the upper limit were diluted 1:40 and reanalyzed. The assay used measured total PCSK9 in serum, so it was not possible to distinguish between bound and unbound PCSK9.

### 2.4. Extraction of Circulating RNAs from Plasma Samples and Reverse Transcription

Circulating RNAs were extracted from 600 µL plasma samples. We used the NextPrep™ Magnazol™ cfRNA Isolation Kit (PerkinElmer, Waltham, MA, USA) as instructed by manufacturer. We eluted the circulating RNAs in 18 µL of RNA Elution Solution (0.1 mM EDTA). To avoid RNA decay, we added RiboLock RNase Inhibitor (Thermo Fisher Scientific, Waltham, MA, USA). We stored isolated circulating RNA samples at −80 °C in LoBind DNA tubes (Eppendorf, Hamburg, Germany). 

RNAs were transcribed into cDNA with the miRCURY Locked Nucleic Acid (LNA) Universal RT Kit (Qiagen, Hilden, Germany). The amount of input RNA was optimized using three different volumes of RNA sample, as recommended by the manufacturer. The volume 0.24 µL was selected as optimal for cDNA synthesis. The reaction mixture contained the following: 5× miRCURY RT SYBR^®^ Green RT Reaction Buffer (2 µL), RNase-free water (6.6 µL), 10× miRCURY RT Enzyme Mix (1 µL), and previously isolated template RNA (0.4 µL). The reverse transcription was carried out using a GeneAmp^®^ PCR System 9700 thermocycler (Applied Biosystems, Waltham, MA, USA). cDNA was stored in a LoBind DNA plate (Eppendorf, Hamburg, Germany) at 4 °C and analyzed within 4 days.

### 2.5. Quantification of miRNA Expression by Quantitative PCR

A literature search was performed to identify miRNAs previously associated with regulation of PCSK9 levels. Five miRNAs were selected for analysis. Expression of the selected miRNAs was measured by quantitative PCR (qPCR). We used specific miRCURY LNA miRNA PCR Assays (Qiagen, Hilden, Germany) (Table 1). Each sample was analyzed in triplicate. cDNA was diluted 1:30 with RNase-free water, except for miR-483-5p, which was diluted 1:10. For each reaction, we prepared a reaction mixture consisting of 2x miRCURY SYBR^®^ Green Master Mix with added low ROX Reference Dye (5 µL), PCR primer mix (1 µL), RNase-free water (1 µL), and cDNA template (3 µL). Each plate also contained a no-template control in triplicate. The qPCR reaction was carried out on a QuantStudio 7 Flex Real-Time PCR System (Applied Biosystems, Waltham, MA, USA) using the following protocol: 2 min at 95 °C; 40 cycles of 10 s at 95 °C, 1 min at 56 °C. The mean cycle of quantification (Cq) values and melting curves for each miRNA were obtained from the instrument software. miR-16-5p and miR-4516 were used as reference miRNAs [24,25]. Relative expression was calculated as 2^−ΔCq^, where ΔCq = average Cq (target miRNA) − average Cq (reference miRNAs) [26].

### 2.6. Statistical Analysis

We used IBM SPSS Statistics version 27.0 (IBM Corporation, New York, NY, USA) to perform the statistical analyses. For descriptive statistical analysis, we analyzed the normality of the distribution of continuous variables with the Shapiro–Wilk test. We used the median with interquartile range (25–75%) or the mean with standard deviation to describe the central tendency and variability of the continuous variables. We used frequencies to describe the distribution of the categorical variables. The Chi-square test was used to compare the distribution of the categorical variables between different groups, whereas the t-test or nonparametric the Mann–Whitney U test was used for the continuous variables. The Wilcoxon signed rank test was used to assess changes during the placebo and treatment periods. Linear regression was used to test the association between PCSK9 levels and expression of miRNAs at enrollment. Correlations between continuous variables were calculated using Spearman’s Rho coefficient. All statistical tests were two-sided. We used 0.05 as the level of statistical significance. Figures were generated using GraphPad Prism 9 (San Diego, CA, USA).

## 3. Results

### 3.1. Baseline Characteristics of Study Subjects

Patients and control subjects were age- and sex-matched and did not differ significantly in systolic and diastolic blood pressure. However, patients had significantly lower total cholesterol, non-HDL-C, LDL-C, apoB (all *p* < 0.001), HDL-C (*p* = 0.005) and apoA1 (*p* = 0.021). Triglycerides did not differ significantly between patients and control subjects. Lp(a) was significantly higher in patients (*p* < 0.001). Details are shown in Table 2.

### 3.2. Characteristics of Patients after Treatment with PCSK9 Inhibitors

Total cholesterol, non-HDL-C, LDL-C, triglycerides, apoB, and Lp(a) decreased significantly (all *p* < 0.001) after the 6-month period of treatment. Total cholesterol decreased by 40.8 (28.1–45.1)%, LDL-C by 69.7 (59.1–78.3)%, triglycerides by 21.1 ((−1.59)–34.4)%, and Lp(a) by 24.0 (12.2–33.0)%. On the other hand, HDL-C and apoA1 levels increased significantly (*p* = 0.004 and *p* = 0.002, respectively). HDL-C increased by 4.78 ((−3.54)–13.3)% and apoA1 by 3.39 ((−2.34)–9.32)% (Table 3). 

### 3.3. Total Serum PCSK9 Level 

**Figure 1:**
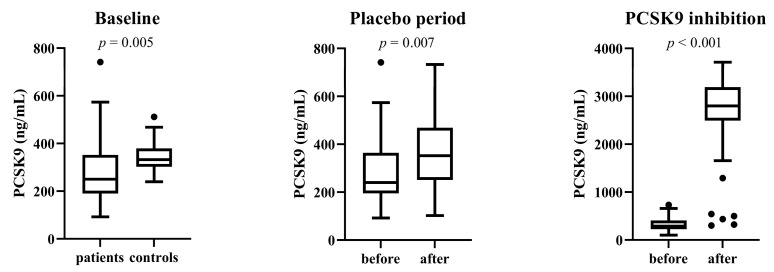
Serum PCSK9 levels in patients and control subjects at baseline, in patients before and after the placebo phase and before and after the 6-month period of therapy with PCSK9 inhibitors.

Patients and control subjects differed significantly in PCSK9 levels (*p* = 0.005). In patients, PCSK9 levels increased significantly after the placebo period (*p* = 0.007). Total serum PCSK9 levels also increased significantly after the 6-month period of treatment. Serum PCSK9 levels are shown in Figure 1.

### 3.4. miRNA Expressions in Patients and Control Subjects before Treatment

**Figure 2:**
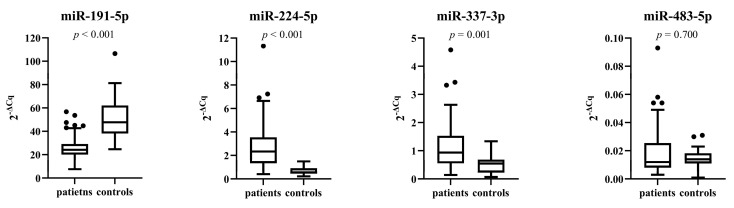
Comparison of miRNAs expression between patients and control subjects.

As shown in Figure 2, the expression of miR-191-5p was significantly downregulated in patients compared with control subjects (*p* < 0.001). Contrary, the expression of miR-224-5p and miR-337-3p was significantly upregulated in patients compared with control subjects (*p* < 0.001 and *p* = 0.001, respectively). We found no significant difference in miR-483-5p expression between patients and control subjects (*p* = 0.700). miR-552-3p could not be determined due to its low expression in plasma.

### 3.5. miRNAs Expression after Placebo and Treatment Period

**Figure 3:**
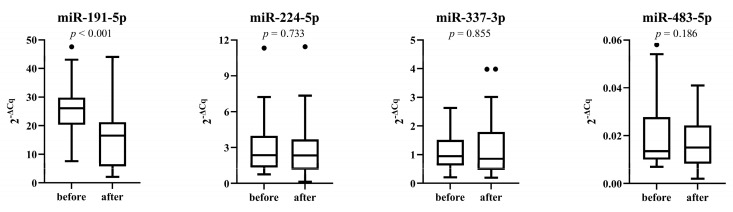
Expression of miRNAs before and after 6 months of placebo phase.

A cohort of 28 patients received placebo for 6 months. Expression of miR-191-5p decreased significantly during the placebo period (*p* < 0.001), whereas expression of other miRNAs did not change significantly (Figure 3).

**Figure 4:**
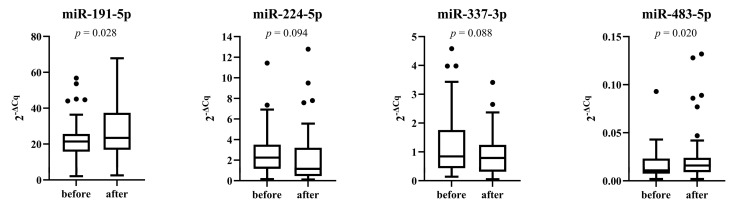
Expression of miRNAs before and after 6-month period of therapy with PCSK9 inhibitors.

The expression of miR-191-5p and miR-483-5p was significantly higher after 6 months of treatment with PCSK9 inhibitors (*p* = 0.028 and *p* = 0.020, respectively). Additionally, the expression of miR-224-5p decreased, but the difference was not statistically significant (*p* = 0.094) (Figure 4). 

### 3.6. Prediction of Serum PCSK9 Level by Circulating miRNAs

A multiple linear regression was performed to predict serum PCSK9 levels based on the expression of the included miRNAs in patients at the time of enrollment. All variables were log transformed because of the skewed distribution. A significant regression equation was found (F (4, 58) = 2.982, *p* = 0.026), with an R^2^ of 0.171. miR-483-5p significantly predicted PCSK9 (β = 0.201, *p* = 0.003), whereas miR-191-5p, miR-224-5p and miR-337-3p did not significantly predict PCSK9 level. Details are shown in Table S1.

Next, a simple linear regression was calculated to predict PCSK9 based on the expression of miR-483-5p. A significant regression equation was found (F (1, 61) = 8.904, *p* = 0.004), with an R^2^ of 0.127. A one percent increase in miR-483-5p expression was associated with about a 0.19 percent increase in serum PCSK9 level (Table S2).

### 3.7. Correlation between the Change of miRNAs Expression and Lipids after Treatment with PCSK9 Inhibitors

**Figure 5:**
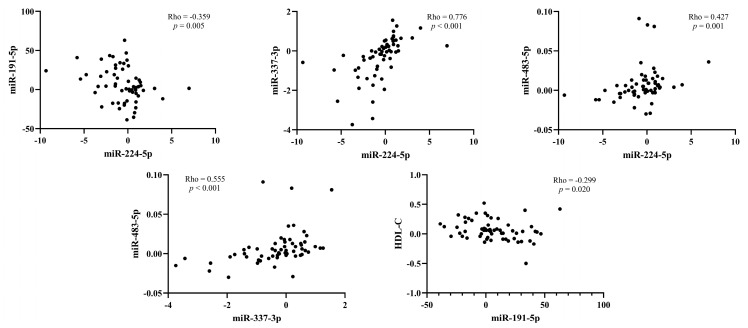
Significant correlations between changes in miRNAs expression and lipid parameters after PCSK9 inhibition. HDL-C, high-density lipoprotein cholesterol.

Spearman’s Rho correlation analyses were performed to investigate possible associations between the change in miRNAs expression, serum PCSK9 levels, and lipid parameters after a 6-month period of therapy with PCSK9 inhibitors. Significant correlations are shown in Figure 5. We found a negative correlation between the change in expression of miR-224-5p and miR-191-5p (Rho = −0.359, *p* = 0.005) and a positive correlation between the change of miR-224-5p and miR-337-3p (Rho = 0.776, *p* < 0.001) and miR-483-5p expression (Rho = 0.427, *p* = 0.001). There was also a positive correlation between the change in miR-337-3p and miR-483-5p expression (Rho = 0.555, *p* < 0.001). The change in serum PCSK9 was not significantly associated with the change in miRNAs expression after treatment with PCSK9 inhibitors. Details are provided in Table S3.

When we evaluated the correlations between the change in miRNAs expression and lipid parameters, including total cholesterol, HDL-C, non-HDL-C, LDL-C, triglycerides and Lp(a), we found a negative correlation between the change in miR-191-5p expression and HDL-C (Rho = −0.299, *p* = 0.020). All results are shown in Table S4. 

## 4. Discussion

Several studies have demonstrated the involvement of miRNAs in regulating cholesterol homeostasis [27], but the regulation of PCSK9 by miRNAs remains poorly understood. Most studies used *in vitro* or animal models, whereas studies in human samples are rare. In the present study, we aimed to investigate the regulation of total serum PCSK9 levels by circulating miRNAs in a well-defined cohort of patients with stable CAD and very high Lp(a) levels. In addition, the expression of miRNAs and lipid parameters were measured after a 6-month period of therapy with PCSK9 inhibitors. Because all patients received statin in maximum tolerated dose to reduce the risk of recurrent cardiovascular events, total cholesterol, LDL-C, and apoB were significantly lower in patients compared with control subjects. However, HDL-C and apoA1 were significantly higher in control subjects. We selected only patients with highly elevated Lp(a) levels; therefore, patients had significantly higher Lp(a) levels. Treatment with PCSK9 inhibitors significantly lowered Lp(a) levels, total cholesterol, LDL-C, triglycerides, and apoB, whereas levels of HDL-C and apoA1 increased significantly.

Serum PCSK9 levels were higher in control subjects than in patients, which is surprising at first glance because they have been associated with the severity of CAD and vascular inflammation [28,29]. Moreover, statin treatment activates the transcriptional activity of sterol regulatory element binding protein-2 (SREBP2), increasing the expression of LDLR and PCSK9 [30]. Indeed, all included patients received statin therapy in maximum tolerated dose. Nevertheless, the variability of PCSK9 levels was markedly higher in patients, whereas PCSK9 levels were more homogeneous in control subjects. In addition, the cohort of control subjects in our study was relatively small because of the strict inclusion criteria. PCSK9 levels also increased significantly during the placebo period although the therapy patients received did not change. The immunoassay used in our study measured total circulating PCSK9, i.e., unbound PCSK9 and PCSK9 bound to therapeutic antibodies. As shown previously, total PCSK9 levels increase after treatment with PCSK9 inhibitors, possibly due to a large increase in PCSK9 bound to therapeutic antibody. Indeed, circulating PCSK9 remains bound to the therapeutic antibody for 2 to 3 weeks [31]. Nevertheless, total serum PCSK9 levels increased approximately 10-fold after 6 months of treatment with PCSK9 inhibitors, similar to previous studies [31,32]. Studies measuring only free circulating PCSK9 (without inactive PCSK9 bound to the therapeutic antibody) showed a decrease in PCSK9 levels after treatment with PCSK9 inhibitors [33]. Recently, preliminary results from the ALIROCKS study reported that total plasma PCSK9 levels could serve as a biomarker for adherence to treatment with PCSK9 antibodies. They suggested that in non-adherent patients, LDL-C levels should decrease by no more than 25% and total PCSK9 levels should increase by no more than threefold after treatment with PCSK9 inhibitors [32]. Based on these criteria, we identified three non-adherent patients in our cohort. Identification of patients with reduced or even absent lipid-lowering response is important because of the costliness of treatment with PCSK9 inhibitors. 

In this study, we focused on the expression of five miRNAs that have previously been reported to interact with and regulate the expression of *PCSK9* mRNA. In addition, miR-224-5p, miR-337-5p, miR-483-5p, and miR-552-3p were found to decrease LDL-C levels in murine models [34,35,36,37,38,39]. miR-552-3p was reported to regulate PCSK9 levels in the human hepatocellular carcinoma cell line (HepG2) [34]. However, we could not clearly determine the level of miR-552-3p in plasma because its expression was very low. Therefore, its contribution to the regulation of serum PCSK9 in humans is questionable and the role of other miRNAs may be more prominent. Moreover, HepG2 cells are not involved in the atherosclerotic process *in vivo*, which could also be a reason for the discrepancies.

None of the above studies examined the difference between patients and control subjects. In our study, we showed decreased expression of miR-191-5p and increased expression of miR-224-5p and miR-337-5p in patients compared with control subjects, indicating their possible involvement in the pathophysiological processes of CAD. The decreased expression of miR-191-5p in patients may be due to the fact that all patients were treated with acetylsalicylic acid and more than half also received another antiplatelet drug (clopidogrel, ticagrelor, or prasugrel). miR-191 is a platelet-derived miRNA, and antiplatelet drugs have been shown to reduce miR-191 expression. In addition, more aggressive antiplatelet treatment decreased miR-191-5p expression more than acetylsalicylic acid treatment alone [40]. Decreased expression of miR-191 was found in patients with acute myocardial infarction compared with control subjects, which returned to the value of controls within 48 h [41] and in patients who experienced major adverse cardiovascular events compared with matched subjects who had no adverse cardiovascular events during the 2-year follow-up period after ST segment elevation myocardial infarction treated with endovascular revascularization [42].

Although miR-483-5p expression was not significantly different between patients and control subjects, miR-483-5p expression was significantly associated with serum PCSK9 levels in patients at baseline. This finding suggests that miR-483-5p is involved in the post-transcriptional regulation of PCSK9 expression *in vivo*. However, in contrast to the results in HepG2 cells [36], our results showed a positive association between serum PCSK9 level and miR-483-5p expression. Nevertheless, miR-483-5p was associated with cardiometabolic risk factors at baseline and during the 3.7-year follow-up period with new-onset diabetes and CAD [43,44]. Increased serum miR-483-5p expression was also reported in patients with asymptomatic carotid artery stenosis compared with controls. At the same time, serum miR-483-5p expression was an independent predictive factor for the occurrence of an acute cerebrovascular event in patients with asymptomatic carotid artery stenosis [45]. 

During the placebo period, miR-191-5p expression decreased significantly, which is rather unexpected. To our knowledge, the effect of treatment with PCSK9 inhibitors on the expression of the included miRNAs has not been investigated. In our study, the expression of miR-191-5p and miR-483-5p increased significantly after 6 months of treatment with PCSK9 inhibitors. Decreased expression of miR-191-5p implies a higher risk of both acute coronary and cerebrovascular events, whereas treatment with PCSK9 inhibitors increased miR-191-5p expression. Based on this, we could propose that one of the pleotropic mechanisms of PCSK9 inhibitors contributing to the reduction of cardiovascular events is the increased expression of miR-191-5p. 

An inhibitory role was attributed to all of included miRNAs in murine models or *in vitro* studies. These results can be partially justified by the fact that each miRNA targets many genes and is therefore involved in different biological processes [17]. For example, miR-224 also targets inducible degrader of the low-density lipoprotein receptor (IDOL), the chaperone protein that promotes lysosomal LDLR degradation, 3-hydroxy-3-methylglutaryl-CoA reductase (HMGCR), the rate-limiting enzyme of cholesterol biosynthesis [35,39], and acyl-CoA synthetase long-chain family member 4 (ACSL4), an essential enzyme in fatty acid metabolism. miR-224 is also a negative regulator of adipocyte differentiation [46]. 

In agreement with previous studies, treatment with PCSK9 inhibitors significantly lowered total cholesterol, LDL-C, triglyceride, and Lp(a) levels and increased HDL-C levels [47,48]. Non-adherent patients were excluded from the correlation analysis. The change in miR-224-5p expression was negatively correlated with the change in miR-191-5p expression and positively correlated with the change in miR-337-3p and miR-483-5p expression. Additionally, there was a positive correlation between the change in miR-337-3p and miR-483-5p expression. This suggests intertwined relationship between these miRNAs. For lipid parameters, we found only a significant correlation between the change in miR-191-5p and HDL-C level after PCSK9 inhibition. Although miR-224-5p, miR-337-5p, and miR-483-5p have previously been shown to lower LDL-C levels in murine models [34,35,36,37,38,39], the change in their expression was not associated with the change in LDL-C level after treatment with PCSK9 inhibitors.

The following limitations should be considered when interpreting our results. First, we included a relatively small cohort of patients, mainly because of the strict exclusion criteria. On the other hand, this resulted in a homogeneous cohort. Second, because of the limited specificity of the ELISA assay, it was not possible to determine the free PCSK9 levels after PCSK9 inhibition, which would be interesting to evaluate possible correlations between the change in free PCSK9 and the miRNAs studied. In this study, we focused only on five previously identified miRNAs that regulate PCSK9 levels. Further studies are needed to comprehensively evaluate the expression of different miRNAs in relation to circulating PCSK9 levels. We must also consider that we do not know to what extent the effects of PCSK9 inhibitors on lipids, especially LDL-C and Lp(a), are responsible for the effects on various miRNAs and how much of the effect can be attributed to so-called pleotropic effects.

In conclusion, our findings demonstrate that miR-483-5p is involved in the regulation of serum PCSK9 levels and therefore may participate in the pathogenesis of CVD. Given the importance of PCSK9 regulation in CVD, miR-483-5p should be considered as a potential biomarker and therapeutic target. Expression of miR-191-5p, miR-224-5p, and miR-337-3p was not associated with serum PCSK9 levels but differed between patients and control subjects and, therefore, may reflect other biological processes in CVD. Treatment with PCSK9 inhibitors increases miR-191-5p expression, which may contribute to the effect of PCSK9 inhibitors on reducing the incidence of cardiovascular events. To our knowledge, our study is the first to investigate the relationship between PCSK9, miRNAs, and lipid parameters. Therefore, this area is clearly understudied, although results from *in vitro* studies and studies in animal models are promising. Further studies are needed to translate this knowledge into the clinic.

## Figures and Tables

**Table 1:** miRNAs, their sequences, accession numbers, and GeneGlobe IDs by Qiagen (Hilden, Germany).

microRNA	Sequence	Accession Number	GeneGlobe ID
hsa-miR-191-5p	5′CAACGGAAUCCCAAAAGCAGCUG	MIMAT0000440	YP00204306
hsa-miR-224-5p	5′UCAAGUCACUAGUGGUUCCGUUUAG	MIMAT0000281	YP02119313
hsa-miR-337-3p	5′CUCCUAUAUGAUGCCUUUCUUC	MIMAT0000754	YP00205938
hsa-miR-483-5p	5′AAGACGGGAGGAAAGAAGGGAG	MIMAT0004761	YP00205693
hsa-miR-552-3p	5′AACAGGUGACUGGUUAGACAA	MIMAT0003215	YP0020603
hsa-miR-16-5p	5′UAGCAGCACGUAAAUAUUGGCG	MIMAT0000069	YP00203906
hsa-miR-4516	5′GGGAGAAGGGUCGGGGC	MIMAT0019053	YP02112882

**Table 2:** Characteristics of patients (N = 64) and control subjects (N = 16) at enrollment.

Parameter	Patients	Control Subjects	*p*
Age at inclusion (years)	52.4 (45.7–56.8)	50.9 (43.5–52.0)	0.207
Gender (% male)	89.1	87.5	1.000
Body mass index (kg/m^2^)	28.7 ± 4.1	26.6 ± 3.2	**0.034**
Systolic blood pressure (mmHg)	129 (120–136)	128 (110–145)	0.813
Diastolic blood pressure (mmHg)	79 (70–82)	77 (68–88)	0.786
Cholesterol (mmol/L)	4.22 ± 0.83	5.46 ± 0.41	**<0.001**
HDL-C (mmol/L)	1.16 ± 0.25	1.52 ± 0.44	**0.005**
Non-HDL-C (mmol/L)	3.07 ± 0.83	3.93 ± 0.55	**<0.001**
LDL-C (mmol/L)	2.29 (1.70–2.66)	3.30 (3.03–3.48)	**<0.001**
Triglycerides (mmol/L)	1.40 (1.00–2.08)	1.28 (0.95–1.51)	0.215
ApoB (g/L)	0.82 ± 0.21	1.07 ± 0.13	**<0.001**
ApoA1 (g/L)	1.28 (1.17–1.44)	1.39 (1.27–1.59)	**0.021**
Lp(a) (mg/L)	1463 (1207–1769)	82 (62–138)	**<0.001**

HDL-C, high-density lipoprotein cholesterol; LDL-C, low-density lipoprotein cholesterol; apo, apolipoprotein; Lp(a), lipoprotein(a). Bold indicates statistically significant values.

**Table 3:** Comparison of laboratory parameters before and after treatment with PCSK9 inhibitors.

Parameter	Before Treatment	After Treatment	*p*
Cholesterol (mmol/L)	4.27 (3.72–4.84)	2.54 (2.10–3.07)	**<0.001**
HDL-C (mmol/L)	1.14 (1.01–1.32)	1.17 (1.02–1.50)	**0.004**
Non-HDL-C (mmol/L)	3.10 (2.40–3.70)	1.20 (0.90–1.78)	**<0.001**
LDL-C (mmol/L)	2.40 (1.75–2.66)	0.63 (0.40–1.15)	**<0.001**
Triglycerides (mmol/L)	1.46 (1.06–2.08)	1.20 (0.78–1.88)	**<0.001**
ApoB (g/L)	0.82 (0.62–0.98)	0.35 (0.35–0.49)	**<0.001**
ApoA1 (g/L)	1.28 (1.19–1.44)	1.35 (1.22–1.54)	**0.002**
Lp(a) (mg/L)	1431 (1219–1776)	1133 (821–1664)	**<0.001**

HDL-C, high-density lipoprotein cholesterol; LDL-C, low-density lipoprotein cholesterol; apo, apolipoprotein; Lp(a), lipoprotein(a). Bold indicates statistically significant values.

## Data Availability

The data presented in this study are available upon request from the corresponding author.

## Supplementary Materials

The following supporting information can be downloaded at: https://www.mdpi.com/article/10.3390/genes14030632/s1, Table S1. Multiple linear regression; Table S2. Simple linear regression; Table S3. Spearman’s Rho correlation between the change in expression of miRNAs after treatment with PCSK9 inhibitors; Table S4. Spearman’s Rho correlation between the change in miRNAs expression and the change in lipid parameters after treatment with PCSK9 inhibitors.
