# Supplementary material for: Interplay between microRNAs, Serum Proprotein Convertase Subtilisin/Kexin Type 9 (PCSK9), and Lipid Parameters in Patients with Very High Lipoprotein(a) Treated with PCSK9 Inhibitors
Source: Genes (Basel). 2023 Mar 3;14(3):632. doi: 10.3390/genes14030632 (PMC10048228; doi:10.3390/genes14030632)
Supplement: Supplementary file 1 [file genes-14-00632-s001.zip › genes-2243815-supplementary.pdf]

genes MDPI

Supplementary Material

Interplay between microRNAs, Serum Proprotein Convertase Subtilisin/Kexin Type 9 (PCSK9), and Lipid Parameters in Patients with Very High Lipoprotein(a) treated with PCSK9 Inhibitors

Tina Levstek, Tina Karun, Andreja Rehberger Likozar, Miran Šebeštjen, Katarina Trebušak Podkrajšek

Table S1. Multiple linear regression.

	Unstandardized β	Coefficients St. Error	Standardized Coefficients β	t statistics	*p*
Constant	20.499	0.240		100.422	**<0.001**
miR-224-5p	−0.005	0.071	−0.008	−0.066	0.948
miR-191-5p	0.205	0.145	0.181	10.418	0.162
miR-337-3p	−0.096	0.069	−0.182	−10.389	0.170
miR-483-5p	0.201	0.066	0.378	30.068	**0.003**

miR, microRNA. Bold indicates statistical significance.

Table S2. Simple linear regression.

	Unstandardized β	Coefficients St. Error	Standardized Coefficients β	t statistics	*p*
Constant	20.762	0.121		220.877	<0.001
miR-483-5p	0.190	0.064	0.357	20.984	**0.004**

miR, microRNA. Bold indicates statistical significance.

Table S3. Spearman's Rho correlation between the change in expression of miRNAs after treatment with PCSK9 inhibitors.

Parameter		miR-224-5p	miR-191-5p	miR-337-3p	miR-483-5p
miR-224-5p	Rho		−0.359	0.776	0.427
	p		**0.005**	**<0.001**	**0.001**
miR-191-5p	Rho	−0.359		−0.111	−0.005
	p	**0.005**		0.399	0.968
miR-337-3p	Rho	0.766	−0.111		0.555
	p	**<0.001**	0.399		**<0.001**
miR-483-5p	Rho	0.427	−0.005	0.555	
	p	**0.001**	0.968	**<0.001**	

miR, microRNA. Bold indicates statistical significance.

Table S4. Spearman's Rho correlation between the change in miRNAs expression and the change in lipid parameters after treatment with PCSK9 inhibitors.

Parameter		Cholesterol	HDL-C	LDL-C	Triglycerides	Lp(a)
miR-224-5p	Rho	0.198	0.214	0.184	0.044	−0.220
	p	0.129	0.101	0.160	0.741	0.097
miR-191-5p	Rho	0.008	−0.299	0.048	−0.028	0.128
	p	0.954	**0.020**	0.713	0.829	0.338
miR-337-3p	Rho	0.188	0.126	0.162	0.142	−0.064
	p	0.145	0.336	0.217	0.279	0.634
miR-483-5p	Rho	0.083	−0.021	0.075	0.183	−0.060
	p	0.531	0.876	0.574	0.164	0.659

HDL-C, high-density lipoprotein cholesterol; LDL-C, low-density lipoprotein cholesterol; Lp(a), lipoprotein(a); miR, microRNA. Bold indicates statistical significance.
